# Moving Together: Understanding Parent Perceptions Related to Physical Activity and Motor Skill Development in Preschool Children

**DOI:** 10.3390/ijerph18179196

**Published:** 2021-08-31

**Authors:** Becky Agard, Nan Zeng, Morgan L. McCloskey, Susan L. Johnson, Laura L. Bellows

**Affiliations:** 1 Department of Food Science and Human Nutrition, Colorado State University, Fort Collins, CO 80532, USA; becky.agard@colostate.edu (B.A.); NZeng@salud.unm.edu (N.Z.); morgan.mccloskey1@gmail.com (M.L.M.); 2 Prevention Research Center, University of New Mexico, Albuquerque, NM 87131, USA; 3 Department of Pediatrics, Section of Nutrition, University of Colorado Anschutz Medical Campus, Aurora, CO 80045, USA; susan.johnson@cuanschutz.edu; 4 Division of Nutritional Sciences, Cornell University, Ithaca, NY 14853, USA

**Keywords:** fundamental movement skills, gross motor skills, parenting, physical activity, preschooler, interviews, qualitative

## Abstract

**Background**: Establishing physical activity (PA) and motor behaviors in early childhood are important for developing healthy activity behaviors. Parents play a central role in shaping young children’s PA and fundamental motor skills (FMS). This qualitative study explored parents’ attributes, values, perceptions, and practices related to PA and FMS. **Methods:** Thirty-one parents (26 mothers) of preschool-aged children participated in semi-structured in-person interviews. Interviews were audio-recorded, transcribed verbatim, coded and analyzed using an iterative approach. **Results:** Four themes related to PA and FMS emerged: (1) parent attributes; (2) parent–child interactions; (3) parent perception of children’s attributes; and (4) parenting practices. Although most parents enjoyed playing with their child, some did not realize the importance of engaging in PA with their child and even believed that FMS are naturally developed. Parents indicated that children’s temperament may influence their preference for practicing motor skills. **Conclusions:** Social support and positive parenting practices, including encouragement, monitoring, logistical support, co-participation, and facilitation, are important for the development of PA and FMS. The findings add emphasis to the importance of parents’ role in the development of young children’s PA and FMS, and they inform future strategies aiming to promote young children’s activity behaviors.

## 1. Introduction

Being physically active at a young age has been associated with numerous health benefits in early childhood and later life, and can lead to a healthy lifestyle for a lifetime [1]. Importantly, activity behaviors are established in early childhood and can track over time [2]. However, only about 50% of U.S. preschool-aged children meet the daily physical activity (PA) recommendations [3], suggesting that this population is at higher risk of acquiring chronic diseases associated with an inactive lifestyle. It is therefore essential to develop healthy activity habits from an early age to position children on a healthy growth trajectory.

Fundamental motor skills (FMS) are a critical aspect of early childhood development, as they are building blocks that lead to specialized movement sequences and sport skills [4]. FMS commonly develop in early childhood and subsequently lead into sport- and context-specific movement patterns that enable lifelong movement experiences [4]. If children receive insufficient FMS instructions and practice during early childhood, they are at higher risk of developmental delays in their motor competence. Although the mastery of FMS has been considered a significant contributor to an active lifestyle [5], only 50% of children have been shown to demonstrate competency in a broad range of motor skills [6]. Since early childhood has been marked as the most intensive period in the establishment of fundamental movement patterns, understanding factors that influence young children’s motor skill development is critical. 

Parents are key contributors to children’s activity behaviors [7]. Specifically, parents influence their children through modeling and the provision of social (e.g., rules and practices) and physical environments (e.g., availability of PA equipment) [8]. Parental influences during early childhood have been shown to be associated with children’s PA in later childhood [9]. Yet, little is known regarding how parents view their preschool child’s PA and their own role in shaping activity behaviors during early childhood. In a study with parents of 5–7-year-olds, participants believed that promoting their child’s PA was unnecessary, as they perceived their child to be sufficiently active [10], suggesting that parents’ knowledge about their child’s PA development is less than optimal. In addition, existing evidence for how best to involve parents to promote their child’s FMS is inconsistent and limited. This is echoed by a recent review study that appealed for more research to gain a comprehensive understanding of parents’ perspectives on their child’s activity behaviors in early life and recognized the important role they play for child development [11]. These findings, along with the limited literature on parents’ views of children’s PA and FMS, highlight the need to better understand parents’ perceptions of their child’s PA and FMS development. 

Over the past two decades, PA-related parenting research has proliferated, with findings confirming the influential role that parents play in children’s activity behaviors [12,13]. This knowledge, however, is yet to be translated into effective family-based PA interventions. The observed research-to-practice gap may be partially explained by limited understanding of structural and contextual dimensions of PA-related parenting [14]. To this end, Davison et al. [14] developed the “Integrated Model of PA Parenting” to frame PA parenting research, and to encourage the exploration of multidimensional PA parenting practices. Specifically, the frame proposes that social-ecological context (i.e., community factors, organizational factors, policy, and historical context) could be impacted by parent PA attributes (e.g., parent history of PA, current PA habits, value parent assigns to PA, etc.), parent perception of child PA attributes (i.e., child competence and preference for PA), and PA parenting practice (i.e., responsiveness, structure, demandingness). These factors, together, would yield mutual connections with a child’s PA outcomes (e.g., enjoyment, motivation, self-efficacy, etc.)

While the “Integrated Model of PA Parenting” provides a solid theoretical foundation, this frame has not been utilized to date to inform behavior change programs that target promoting PA parenting practices. As such, more research is encouraged to employ and refine such theories and relevant construct definitions for the application to PA-related parenting. Driven by Davison et al.’s framework [14], the current study aimed to qualitatively explore attributes, values, perceptions, and practices of parents with preschoolers in relation to PA and FMS. The findings of this study will provide unique insight into potential strategies that seek to promote activity behaviors during early childhood.

## 2. Materials and Methods

### 2.1. Study Design

This study conducted qualitative, semi-structured interviews as part of the formative research phase of the Healthy Environments (HEROs) study, which aims to promote healthful eating and PA behaviors among preschool-aged children [15]. HEROs focused on families living in rural areas of Colorado, USA, who had a child attending Head Start or Colorado Preschool programs (i.e., federally or state-supported early childhood education programs). The Consolidated Criteria for Reporting Qualitative Research guidelines were followed [16]. The study adhered to the ethical guidelines for human research and was approved by the Institutional Review Board at Colorado State University.

### 2.2. Research Team

The research team comprised 5 researchers (4 female and 1 male) with expertise in PA and FMS development (N.Z., L.L.B), public health (M.L.M., L.L.B.), and child development (S.L.J.). Three team members had expertise in qualitative research (M.L.M., S.L.J., L.L.B.). Two graduate students were involved in the study (B.A. and the interviewer) and were trained on qualitative methodologies. Students were overseen by 2 principal investigators (S.L.J and L.L.B).

### 2.3. Recruitment

Participants were recruited from a large sample of primary caregivers participating in the HEROs study. In the spring of 2016, parent information packets were sent home via children’s backpacks with 775 children attending preschool centers to recruit both fathers and mothers. Of the 119 parents who returned interest forms, 35 parents were randomly selected, and 31 provided informed consent and participated in the interview.

### 2.4. Development of Interview Guide

A semi-structured in-person interview guide was developed to understand parental perceptions of interest. Davison et al.’s [14] “Integrated Model of Physical Activity Parenting” provided a framework for the development of interview questions, specifically the constructs of parent PA attributes, perceptions, and PA parenting practices. Key topics in the interview guide included: (1) parents’ experiences of PA when they were growing up; (2) parents’ current PA habits; and (3) parents’ values and practices related to promoting PA and the development of FMS in their preschoolers. Notably, due to Davison et al.’s [14] model focus on only PA, the current study extended similar constructs to explore FMS parenting behaviors. 

Content validity was established via review by authors with their respective expertise. Face validity was established via pilot testing and cognitive interviews with 4 members of the target audience to ensure terminology was clear and questions resonated with the audience [17]. Subsequently, minor modifications were made to finalize the order, flow and wording of questions (e.g., motor skills were used instead of FMS as the former was a more recognizable term for this audience). The final interview guide consisted of an introduction describing the overall topic and meaning of the term ‘motor skills’, followed by 15 questions with multiple probes. The guide was designed to produce interpretive and descriptive information (Table 1). One notable feature of the interview guide was the inclusion of two vignettes, stories for parents to react to in an attempt to minimize social desirability and to elicit responses that were reflective of parents’ true attitudes and behaviors [18]. 

### 2.5. Data Collection

Interviews were conducted by one trained researcher at participants’ homes or a public setting (e.g., preschool, library), ranged between 50 and 75 min, and all were audio-recorded with participants’ permission. At the start of the session, the interviewer provided an overview of the study, her role in the research and interest in the topic. In some instances, due to the location of the interview, other family members (e.g., children, spouse/partners) were present for the interview but were not active participants. After every 3–4 interviews, research team members reviewed notes and responses per question to ascertain if the type of information received was adequate to answer research questions and to monitor saturation. The a priori expectation was that saturation would be reached with approximately 30 participants. Qualitative studies in which participants possess in-depth knowledge of the topic (e.g., parents responding to questions about their children and personal experiences) tend to reach saturation with smaller sample sizes [19]. Consistent with this expectation, the final interviews provided no additional insights, thus indicating that theoretical saturation had been achieved. Preliminary analysis of the interviews included member checking, in which key points and interpretations from each interview were summarized and sent to the interview participants for feedback. Transcripts were validated by a member of the research team who listened to each audio recording while reading the corresponding transcript to identify discordant phrasing. The recordings were de-identified and transcribed verbatim by a Health Insurance Portability and Accountability Act (HIPPA)-compliant vendor and verified for accuracy. The final transcripts were imported into NVivo (NVivo qualitative data analysis Software; QSR International Pty Ltd., Burlington, MA, USA, Version 11, 2015).

### 2.6. Data Analysis

A directed approach to qualitative content analysis was used to analyze the interviews [20]. The preliminary codebook was based on constructs from Davison et al.’s model [14], with codes added to capture content that emerged in the interviews that were not part of the model, namely, FMS-related codes. The preliminary codebook was developed by two trained researchers who read the interview transcripts several times and each created codes and definitions organized by overarching category.

To refine code definitions and inclusion/exclusion criteria, the same two trained researchers coded two interview transcripts together based on the qualitative training protocol by Goodell et al. [21]. Thereafter, the two coders independently coded nine additional transcripts and established strong interrater reliability (kappa = 0.93). The coders divided the remaining transcripts, with each reviewing the coding of the other. Discrepancies were resolved through discussions with a third senior member of the research team. To interpret the results, two researchers independently read through all quotes associated with each code and each identified key themes. The researchers met several times to achieve consensus on derived themes that were associated with each code and that emerged throughout the entire data set [20].

## 3. Results

Among the 31 participating parents, 26 (84%) were mothers; 11 (35%) identified as Hispanic; 10 (32%) had a high-school diploma or less; 20 (65%) were considered low income, defined as 185% of the U.S. federal poverty level [22]; and 18 (58%) of the preschoolers discussed were boys. Data were grouped into four themes related to the social context of young children’s PA and FMS: (1) parent attributes, (2) parent–child interactions, (3) parent perception of children’s attributes and (4) parenting practices. Themes are presented below by category and exemplar quotes are provided in Table 2.

### 3.1. Parent Attributes

#### 3.1.1. Parent History of PA

When parents considered their own PA as children, it was primarily playing outside to “*hangout out with friends.*” For parents who reported participating in organized sports, the primary reason for doing so was because they were “*good at it*” and that made it fun. The opposite was true for parents who avoided organized sports. Several parents mentioned that they “*hated running*” or were “*afraid of the ball*”, which prevented them from playing sports. For most parents, they enjoyed playing outside and playing with friends, and considered it “*something to do to pass the time*.”

Notably, parents indicated that social support was an important factor in their own childhood participation in PA. Specifically, most parents did not report playing with their own parents but did remember that their parents both enabled them to play sports and attended their games. Several parents remembered a specific person who was instrumental in their PA habits by igniting their interest in a particular sport, practicing skills with them, or just providing ample opportunities for them to be physically active. Conversely, parents who did not participate in sports or play much as they got older felt it was because they lacked someone to encourage them. 

#### 3.1.2. Current PA Habits

Most parents indicated that they did not have an *exercise routine*, and therefore they considered playing with their children as their primary way of exercising. In addition to playing with their children, parents mentioned going on walks, physically demanding jobs, and engaging in workouts at home. While some parents noted that they exercise regularly because this was good for their own mental and physical health benefits, lack of time, family duties, physical limitations, and being out of shape were cited as barriers for parents to participate in PA.

### 3.2. Parent–Child Interactions

#### 3.2.1. Value Parent Assigns to PA

Parents valued participation in PA for both child-centric and self-centric reasons. Specifically, they wanted their children to be active for health reasons that included “*prevent diabetes*,” not being “*overweight*,” “*building up their muscles,*” because it is “*good for your heart*” and just “*to be healthy.*” Parents also described exercise as a “*stress relief,*” improving their “*self-esteem,*” a way to “*lose weight*” and “*be healthy*” for themselves. Meanwhile, they considered PA as having a positive impact on “*good hand-eye coordination,*” teaching children to “*pay attention,*” enabling children to “*gain confidence*” and “*improve self-esteem*.” Parents appreciated the social aspect of PA in that it provided a medium for family bonding and the development of child friendships. 

Moreover, parents viewed being “*active*” as a positive personal trait that they wanted their children to embody as children and throughout life. Parents wanted their children to participate in a variety of activities, and thus they enabled them to find one that they liked. Parents also encouraged their child’s participation in PA as a tool for behavior management. Parents reported that when their child participated in PA, it “*makes bedtime easier*,” “*gets their energy out*” so they are “*easier to manage*,” and “*keeps them from getting in trouble*.” Parents also suggested that encouraging their child to be active made them happy because they enjoy seeing their child happy, growing, learning and having fun, and it made them feel like good parents. It is noteworthy that despite most parents’ realization of the importance of playing with their child, some mentioned they were distracted while interacting with their child. Consequently, they would let the child play alone to give the parent “*a break*” or to give them time to “*get chores done*” or simply “*relax.*”

#### 3.2.2. Value Parent Assigns to FMS

Parents had different perspectives about the importance of their children developing FMS. Several parents suggested that hand–eye coordination, running and balance were specifically important skills to have to get through life. Some parents felt that ball skills were important for their children’s future participation in sports. Although these parents indicated that FMS were an important “*part of life,*” they failed to provide specific reasons why FMS were important. Further, some parents did not see FMS as important and assumed that children will learn skills as the need arises.

#### 3.2.3. Parent Self-Efficacy to Teach FMS

Although several parents felt confident in their ability to teach basic FMS to their child, they did not specify how they were doing this. Notably, some parents expressed concern that they would not know how to teach “*sport skills*” and said, when the time came, they would ask someone else or enroll their child in sports, so they could learn from a coach. Other parents reported specific instances of teaching their child a particular skill, and this was typically prompted by noticing that their child was struggling with a skill or that their child needed to learn a skill, so they could play a game that the family was playing. Lastly, a few parents stated that they did not feel the need to teach motor skills while playing.

### 3.3. Parent Perception of Child Attributes

Parents had clear perceptions of their child’s competence and preferences for PA and FMS. Specifically, some parents viewed their children as having locomotor competence, except for skipping, which parents of boys indicated their child had not fully mastered. These parents also reported ball skills as needing the most improvement in terms of catching, throwing and kicking with appropriate speed and direction. Parent perception of less-developed ball skills was more prominent with parents of girls. It was reported that most children enjoyed PA, whereas preference for practicing motor skills varied by temperament. According to parents, some children were more eager to learn new skills and determined to practice until they felt competent, whereas other children were reported to have a hard time venturing out of their comfort zones to play new games or try new skills. These children were reported to get frustrated and quit when they could not perform a skill. 

### 3.4. Parenting Practices

#### 3.4.1. Responsiveness

Parents used responsive practices such as offering praise and engaging in child-driven play to foster enjoyment and motivation for PA. Parents frequently used reasoned communication to promote the development of FMS. For instance, parents encouraged children to try things and to *“do your best,”* advised children that “*the more you practice, the better you’re going to get,”* and reminded children that *“when you go get out there, all your friends that are your age, they’re learning, too.”* While their children were learning motor skills, parents frequently reported offering warmth in the form of encouragement and praise for both trying and succeeding at the skill. In addition, parents used autonomy support to create positive PA experiences by engaging in child-driven play at home.

#### 3.4.2. Demandingness

Parents frequently reported telling their children to *“go outside and play,”* as an alternative to the child engaging in technology. Parents also set rules that restricted PA to certain areas to protect the house and the safety of the child. Few parents used PA/playing as a reward after children completed their chores. Some mothers reported fathers placing maturity demands on their children by expecting them to be more advanced in motor skills than they should be for their age.

#### 3.4.3. Structure

Parents attempted to provide sports equipment at home to create an environment conducive to learning FMS. Alternatively, they took their children to a place to play, such as a park, open space, or a family member’s house. In general, most parents gave their children opportunities to play either by signing them up for sports or playing games at home or with the family. Parents also built a social environment that facilitated the development of FMS by playing with their children or playing games with their extended family. They cited the lack of equipment, space, opportunities and social support as barriers to children participating in PA and learning FMS. 

## 4. Discussion

Parents serve as role models for activity behaviors, making them crucial contributors to their children’s healthy lifestyles. Therefore, increased knowledge of parenting in relation to PA and FMS during early childhood is especially important in understanding how best to help and support parents to promote their child’s health behaviors, prevent/reverse childhood obesity and minimize other adverse outcomes. This study sought to comprehensively understand parents’ attributes, values, perceptions, and practices related to PA and FMS—setting the stage for the development of intervention strategies to promote improvements in preschool children’s activity behaviors and FMS development. 

Parental attributes from their own activity history and habits are important for the development of a child’s activity behaviors [14], as active parents are more likely to proactively promote their child’s PA [23]. In this study, parents reported that when they were a child, they played sports because they were good at it (i.e., possessed competence). Conversely, some chose not to play sports because they felt little confidence in their abilities, suggesting that a lack of physical self-competence could prevent an individual from participating in PA. This is consistent with review evidence that motor skill competence is positively associated with PA in children [24,25], highlighting the importance of the development of FMS during early childhood [26]. The importance of social support for participating in PA was exemplified by parents reporting that they played sports because they were encouraged and motivated by their parents/friends, even when parents/friends were not actually playing with them. Previous studies revealed that parents/friends supporting, watching, and praising children was positively related to youth PA [27]. As such, social support is a significant contributor to the development of children’s activity behaviors. 

Parents valued PA for both child-centric and parent-centric reasons. While most parents reported enjoyment when playing with their child and considered PA a gateway for their child to have active lives that include good health and parent–child interaction, several parents viewed PA as a distraction when interacting with their child, and thus let the child play alone so they could take a break or get chores done. These opposing perspectives could partially explain the inconsistency in the literature regarding the relationship between parent PA and child PA [28]. Previous evidence noted that possible mechanisms for the association between parents’ and their child’s activity levels include the sharing of activities by family members [29]. A recent study reported that parental participation in PA may be an important component to maximize PA behaviors in preschool children [30]. Parental influence on a child’s PA can be small if parents and children are not consistently engaging in PA together. Encouragement of parental engagement in PA with their child may be mutually beneficial at increasing PA in both groups.

Overall, participants’ knowledge of FMS was limited. While some parents suggested that FMS were important for their child’s life, none mentioned ways in which FMS are related to PA. Specifically, a few parents believed that their child was naturally proficient in motor skills and thus there was little need to teach and help their child master these skills. Our findings correspond with the results of a previous qualitative study in which parents reported little active promotion of PA for their preschool-aged children due to the belief that children naturally achieve sufficient levels of PA [31,32]. The belief that a child is innately skilled in motor competence is at odds with evidence showing that only 50% of young children demonstrate competency in a broad range of motor skills [33]. Therefore, the lack of motor proficiency observed in young children may partially stem from parents’ knowledge gap and lack of awareness of the development and progression of young children’s FMS. This is underscored by parents’ expressions of concern that they lacked self-efficacy and the ability to teach motor skills. Indeed, young children need stimulation and opportunities to help them practice and master various motor skills, both simple and complex. The misconception that children are naturally motor proficient has important implications for the development of intervention strategies aimed at facilitating children’s FMS. If parents do not realize the importance of their role, it is unlikely that they will engage in strategies to promote their child’s FMS. 

Interestingly, parents indicated that children’s preference for practicing motor skills varied by child temperament. For example, some children were more eager to learn new skills and determined to practice until they felt competent. Conversely, a few children were reported to have a hard time venturing out of their comfort zones to learn new skills. Consequently, these children tended to get frustrated and quit when they cannot perform a skill. Of note, psychologists and pediatricians have demonstrated that children who are mildly active, rhythmic, adaptable, persistent, and approachable attain a more advanced developmental level (i.e., cognitive-verbal and motor skills) during their preschool years [34,35]. This knowledge, to date, has not yet been applied to effective strategies and programs that target children’s motor skill development. Most behavioral interventions underestimate or ignore the importance of children’s temperament for the development of motor behaviors, and therefore they may fail to consider children’s temperament as a critical intervention component. Future studies need to further understand the relationship between temperament and activity behaviors in early childhood and include temperament as a key consideration in tailoring PA interventions to promote young children’s motor skills.

Activity-related parenting practices refer to ways in which parents shape their child’s activity behaviors [36]. Considering that PA and FMS are necessary components of activity behaviors, elements of the social context in which children participate in PA and learn FMS are likely to have an impact on children’s health behaviors. Parents generally were responsive to the idea of promoting their child’s PA and FMS. For instance, parents indicated that they would motivate the child to try new things (e.g., kicking a ball) by offering warmth in the form of encouragement and praise for both trying and succeeding, as well as engage in child-driven active play to create a positive PA experience for their child—all attributes of responsive parenting. In the context of play, responsive parenting reflects reciprocity between child and parent. Previous literature documented that children’s PA levels are strongly associated with parent encouragement and would be promoted by positive PA experience [37], suggesting that parents’ responsive behaviors may promote advances in child development. 

In the current study, parents reported that they would intentionally monitor their child’s activity behaviors by setting limits on sedentary pastimes and promoting activity. Although PA parenting practices reflecting demandingness or control, to some extent, are associated with negative PA outcomes, such as decreases in children’s enjoyment of PA [38], parental monitoring of PA may also lead to increasing children’s PA levels [39], thereby creating more opportunities for children’s development of FMS. Of note, some mothers indicated that fathers placed high maturity demands on their child’s physical activities, highlighting other demandingness practices. 

It was generally recognized that the structure domain, including logistic support, such as providing sports equipment and creating a safe environment, as well as co-participating and enrolling children in organized PA could be beneficial to promoting children’s PA and FMS. For example, parents suggested that they would attempt to create an environment conducive to the development of PA and FMS by providing sports equipment at home, taking their children to park, and signing them up for sports. This finding mirrors previous evidence that logistic support could help children establish early activity patterns [40,41]. Co-participation and facilitation were also significant constructs for the structure domain along with PA parenting practices [42], indicating the importance of these factors in the development of children’s PA and FMS. 

This study is not without limitations. Despite our study seeking to investigate PA-related attributes, values, perceptions, and practices of both parents, few fathers participated, and thus the PA and FMS practices of fathers are less clear and represent an opportunity for future research. Indeed, the dilemma of how to effectively recruit both parents to participate in research has been noted by other studies attempting to recruit and engage fathers of young children in research. The small sample has low cultural, economic and geographic diversity; thus, generalization of the results to parents from different backgrounds is limited. Nevertheless, this study has its strengths. The interview guide was based on a theoretical model and was assessed for both face and content validity. Strong interrater reliability was established between coders. 

By investigating parents’ attributes, values, perceptions, and practices in relation to FMS development, this study provides meaningful information for the future intervention strategies aiming to promote young children’s FMS. The findings suggest that the development of FMS is a more complex process, involving multiple unique parenting practices such as communicating with children about the importance of trying and practicing new skills, providing equipment that enables children to use movement skills, and using creative ways to teach children movement skills. These constructs should be considered in future theories and models applying PA- and FMS-related parenting to young children.

## 5. Conclusions

In this study, parents of preschool children lacked knowledge and awareness in relation to their child’s development of PA and FMS, and thus they may have overlooked the importance of helping their child establish early activity patterns. Addressing the erroneous parental perception that children innately develop motor skills has important implications for the development of intervention strategies facilitating children’s FMS. Moreover, future strategies aiming to promote young children’s FMS may need to consider temperament as a strategy to tailor PA interventions. Lastly, social support and positive parenting practices related to PA, such as encouragement, monitoring, logistical support, co-participation, and facilitation, may contribute to promoting improvements in preschool children’s PA and FMS. Hence, involving parents in children’s daily PA and incorporating social support and positive parenting practices may enrich future parent–child interactions to increase children’s PA and FMS.

## Figures and Tables

**Table 1:** Sample questions by construct asked in semi-structured, in-person interviews with parents of preschoolers about physical activity and fundamental motor skills.

Constructs	Overarching Questions	Probes
Parent history of PA	Tell me what physical activities were like for you growing up; this can be anytime during your childhood, not just during preschool. Overall, how important would you say physical activity was to your family’s life?	What types of physical activity did you do? What about being active was/was not important to your family? You said physical was [answer from previous probe] important to your family’s life. Does that apply to everyone or only to some people in your family?
Parent current PA habits	Which physical activities do you do now?	What are the reasons you don’t do [activity]?
Parent perception of child PA/FMS attributes	Can you tell me about any sport- or motor-related activities that [child’s name] currently does? As a reminder, ‘motor skills’, are skills preschoolers are learning to do, like being able to catch/throw/hit a ball, or leap/hop/skip.	Tell me more about that. Are there any other motor-related activities [child’s name] does?
Parent perception of child PA/FMS attributes	Are there any types of sports or motor skills you wish [child’s name] could do that he/she can’t do yet?	Tell me more about that Are there certain motor skills you think are important for [child’s name] to learn? Please explain. (If participant talks about riding a bike)- What about riding a bike is important to you for [child’s name] to be able to ride a bike?
Value parent assigns to PA and FMS	What are some of the reasons you have [child’s name] do physical activities?	Would you explain that a little more? Why is it important to you? What do you think it does for your child? What do you think it does for you?
Value parent assigns to PA and FMS Parent self-efficacy to teach FMS Parenting Practices	Vignette: Let’s think about a few years from now. There is a little boy/girl named Alex(a), (s)he is a first grader. During recess at school, a group of Alex(a)’s classmates decide to play kickball. Alex(a) kind of wants to play but decides not to because (s)he’s not very comfortable with how well (s)he kicks a ball, so (s)he doesn’t want to do it in front of the other children. Instead, Alex(a) plays on the playground equipment.	What do you think Alex(a)’s parents could/should do about him/her not feeling very comfortable with how well (s)he can kick a ball? Are there any other things you think parents might be able to do to help their preschooler learn different motor skills? Please explain. (What makes you think that?) What are some reasons you think it might matter whether or not a child knows how to kick a ball or knows how to do other motor skills? –We use kicking a ball in this example, are there ball skills other than kicking a ball that you think are important?

**Table 2:** Themes and participant exemplar quotes related to physical activity and fundamental motor skills of preschoolers.

Themes	Subcategories	Exemplar Quotes
Parent attributes	Parent history of PA	“*They would take me to my practices and my track meets and my games, and they would always come and stay. There were times they would come and just watch practice just to give me that little motivation*.” “*I just never did it. I guess I needed somebody to really push me to it, because I really liked the sport, but I just didn’t want the commitment*.”
	Current PA habits	“*I don’t do a whole lot of anything right now. The most I do is I walk to church on Sundays, so the family usually takes the vehicle and stuff and I walk*.” “*I do a lot of walking with the children. We do go a lot to the park. Mostly on the weekends*.” “*There is no time, and then the time that there is you just want to try and rest*.”
Parent–child interactions	Value parent assigns to PA	“*I think just being able to socialize with friends. There’s a lot of children that just walk around the playground, they look like they don’t have any friends because they maybe don’t actually know how to play any of those sports*” “*It makes me very proud of them. It makes me happy to see him happy. It makes me feel like a better parent. I know that he’s been real active. When I see that he’s tired it’s like I did my job*.” “*Sometimes I do have a lot on my mind and I don’t want to focus on playing and I don’t want to be busy with that*.”
	Value parent assigns to FMS.	“*It’s not really important if they do or if they don’t know how to kick a ball*.” “*When I think of motor activities, I always think sports. I do want my children, once they get older, to be involved in a lot of sports just to stay active*.” “*You might not necessarily use it as an adult, but as a kid, playing together with balls and running around and stuff is a really big social thing*.”
	Parent self-efficacy to teach FMS	“*I think I am not necessarily teaching her unless she takes the initiative to try and do something, or I see she is struggling*.” “*I mean I’m just having fun. I didn’t think about teaching her motor skills saying like tuck and roll or kick a ball. It’s just like a game. They’re learning from it, but I don’t really think about it like that*.” “*So they’re kind of just doing stuff that they know how to do and stuff they can teach themselves to do*.”
Parent perception of children’s attributes		“*She is very determined, she is very independent. Even if I try to teach her something, she would try and try until she got it right*.” “*When he gets frustrated, sometimes he just doesn’t want to do it anymore*.”
Parenting practices	Responsiveness	“*Any sort of success I think should be celebrated, especially when a child is uncomfortable with their competence and their skills*.” “*Just whatever he feels like doing he’ll ask us, “Will you do this with me?” and we always tell him okay*.”
	Demandingness	“*I do let them play on their tablets and watch TV, but then I’m like, “All right, you guys need to go play outside too*.” “*We are on the second floor, so we try to keep inside activity limited. We don’t send her outside by herself*.”
	Structure	“*In our household it’s hard to pay attention to one kid at a time to do something with because there are six of them in the house, so it’s very rare that one of us will get one-on- one time with the other children*.”
